# Binding to the neonatal Fc receptor enhances the pathogenicity of anti-desmoglein-3 antibodies in keratinocytes

**DOI:** 10.3389/fimmu.2024.1473637

**Published:** 2024-10-10

**Authors:** Anna Zakrzewicz, Katrien Vanderheyden, Yad Galaly, Simon Feldhoff, Magdalena Sips, Maximilian Brinkhaus, Ritva Tikkanen

**Affiliations:** ^1^ Institute of Biochemistry, Medical Faculty, University of Giessen, Giessen, Germany; ^2^ argenx, Ghent, Belgium

**Keywords:** autoimmune diseases, autoantibodies, bullous skin diseases, pemphigus, neonatal Fc receptor, Fc fragment, desmoglein 3

## Abstract

The neonatal Fc receptor (FcRn) is important for numerous cellular processes that involve antibody recycling and trafficking. A major function of FcRn is IgG recycling and half-life prolongation, and FcRn blockade results in a reduction of autoantibodies in IgG-mediated autoimmune diseases. In epithelial cells, FcRn functions in processes different from IgG recycling, such as antibody transcytosis in intestinal cells. In pemphigus vulgaris, an autoimmune disease of the epidermis, IgG autoantibodies directed against desmosomal adhesion proteins, especially desmoglein-3 and -1, cause loss of keratinocyte adhesion. We have previously demonstrated that FcRn blockade with efgartigimod, a human Fc fragment with enhanced FcRn binding, significantly reduces the keratinocyte monolayer fragmentation caused by anti-desmoglein-3 antibodies. This points to a direct function of FcRn in keratinocytes, beyond IgG recycling, but the mechanisms have not yet been elucidated in detail. Here, we show that FcRn binding is required for the full pathogenicity of recombinant anti-desmoglein-3 antibodies in keratinocytes, and that antibodies that exhibit enhanced or reduced FcRn affinity due to targeted substitutions in their Fc region, as well as F(ab’)2 fragments not binding to FcRn display different degrees of pathogenicity. Blockade of FcRn by efgartigimod only shows a protective effect on keratinocyte adhesion against antibodies capable of binding to FcRn. Furthermore, antibody-induced degradation of desmoglein-3 in keratinocytes does not depend on FcRn, demonstrating that desmoglein-3 degradation and acantholysis are functionally disconnected processes. Our data suggest that the role of FcRn in autoimmune diseases is likely to be versatile and cell-type dependent, thus stressing the importance of further studies on FcRn function in autoimmune diseases.

## 1 Introduction

The neonatal Fc receptor (FcRn) was originally reported to transport IgG from the mother to the fetus (1). However, further functions of FcRn in various processes and cell types are emerging, such as prolongation of antibody half-life, albumin transport, IgG transcytosis, antigen presentation, and others. The functional aspects of FcRn in these processes have been extensively reviewed elsewhere and are thus not further elaborated here (2, 3).

FcRn is a dimeric receptor consisting of a single-pass transmembrane α-chain and β2-microglobulin that is also found in major histocompatibility complex I (MHC I) (4–7). One of the major roles of FcRn is the recycling of albumin and IgG molecules, but albumin and IgG binding occurs in different regions of FcRn, and may thus take place simultaneously. Each FcRn binds one albumin, whereas two FcRn molecules are engaged in binding one IgG (8–10). Interaction of the Fc region of IgG molecules with FcRn is mediated by the CH2 and CH3 regions of the IgG and takes place in the acidic environment of endosomes (4, 5, 11, 12). Thus, during IgG recycling, FcRn binds pinocytosed IgGs in endosomes and releases them in the extracellular space after transport to the cell surface, resulting in a prolongation of IgG half-life due to FcRn-mediated avoidance of lysosomal degradation. The affinity of IgG to FcRn is regulated by the protonation state of key His residues in the IgG Fc regions, which facilitates the pH-dependent binding (13–15). Therefore, modulation of FcRn affinity of IgG molecules either by antibody engineering based on targeted amino acid substitutions, or by natural Fc variants affects the half-life of the IgG (2, 3, 11, 16). Exchange of three residues in the Fc region, Ile253, His310, and His435 to Ala (IHH variant), abrogates FcRn binding, whereas the substitutions Leu309Asp, Gln311His and Asn434Ser (DHS variant) enhance FcRn affinity at acidic pH (10, 17).

In several autoimmune diseases, including generalized myasthenia gravis (MG), immune thrombocytopenia (ITP), and chronic inflammatory demyelinating polyneuropathy (CIDP), blockade of FcRn results in a reduction of IgG autoantibodies and in clinical improvement of the patients (18–21). One of the FcRn blockers validated in clinical studies is efgartigimod, a human Fc fragment that carries five amino acid substitutions (Met252Tyr, Ser254Thr, Thr256Glu, His433Lys, and Asn434Phe), also known as MST-HN or ABDEG (antibodies that enhance IgG degradation) substitutions (22, 23). Efgartigimod shows an enhanced FcRn affinity at both acidic and neutral pH and thus blocks FcRn-mediated IgG recycling, resulting in reduced half-life of IgG by targeted lysosomal degradation (22, 24).

Pemphigus vulgaris (PV) is a blistering autoimmune disease of the epidermis, characterized by IgG autoantibodies mainly against the desmosomal adhesion proteins, especially desmoglein-3 (Dsg3) and desmoglein-1 (Dsg1). We previously demonstrated that blockade of FcRn by efgartigimod results in protection of the keratinocyte monolayer integrity upon anti-Dsg3-IgG treatment, and this mechanism does not rely on inhibition of antibody recycling (25), originally suggested as the main mode of action of FcRn blockers *in vivo* (22). Therefore, FcRn in keratinocytes may contribute to the local PV pathology in the epidermis in an unexpected manner.

The purpose of the present study was to generate recombinant anti-Dsg3 antibodies that exhibit different binding affinities to FcRn, and to explore if their pathogenicity towards the keratinocyte monolayer is dependent on their FcRn binding propensity. We here show that binding to FcRn enhances the pathogenicity of the anti-Dsg3 antibodies in a manner that is independent of Dsg3 degradation, and that the protective effect of efgartigimod is only observed in the case of antibodies capable of FcRn binding. These findings further support the local function of FcRn in keratinocytes by modulation of PV pathogenesis and support the use of FcRn antagonists in the treatment of PV.

## 2 Materials and methods

### 2.1 Cell culture

The human keratinocyte cell line hTert/KER-CT (Cat. CRL4048; ATCC, Manassas, Virginia, USA) was obtained from LGC Standards GmbH (Wesel, Germany) and cultured as described (25, 26). Briefly, the cells were kept in T-25 cell culture flasks (Sarstedt, Nümbrecht, Germany) in a humidified atmosphere at 37°C, with 5% CO_2_ in basal Keratinocyte Growth Medium 2 (KGM2) with supplements consisting of 4 µl/ml bovine pituitary extract, 0.125 ng/ml recombinant human (rh) epidermal growth factor, 5 µg/ml rh insulin, 0.33 µg/ml hydrocortisone, 0.39 µg/ml epinephrine, 10 µg/ml rh transferrin, and 0.05 mM CaCl_2_ (Cat. C20011; PromoCell, Heidelberg, Germany), as well as 30 µg/ml gentamicin sulfate (Serva, Heidelberg, Germany). For experiments under high calcium conditions, the cells were grown in KGM2 with 2 mM CaCl_2_.

The human acute monocytic leukemia THP-1 cell line (Cat. TIB-202; ATCC) was maintained in RPMI 1640 (Cat. A10491-01; Life Technologies Europe, Bleisweijk, Netherlands) supplemented with 10% fetal bovine serum (FBS, Cat. F7524; Sigma-Aldrich, Darmstadt, Germany), penicillin (100 U/ml), streptomycin (100 µg/ml) (Cat. P4333; Sigma-Aldrich), and 0.05 mM beta-mercaptoethanol (Cat. 31350010; Life Technologies). The cells were grown in a humidified atmosphere with 5% CO_2_ at 37°C.

### 2.2 Antibodies and antibody fragments

Recombinant anti-Dsg3 AK23 IgG variants with human and mouse Fc region were transiently produced in human embryonic kidney HEK293E cells (Thermo Fisher Scientific, Darmstadt, Germany), and purified using MabSelect PrismA, MabSelect Sure LX (both from Cytiva, Chicago, USA), or CaptureSelect Kappa XP (Life Technologies), depending on the Fc variant. This was followed by gel filtration using a Superdex 200 Increase 16/40 or 16/600 column (Sigma-Aldrich). The expression and purification were performed by ImmunoPrecise Antibodies (Utrecht, Netherlands). Human AK23 F(ab’)2 was transiently produced in Expi293 cells (Life Technologies), and purified using KANEKA KanCap™ G (Kaneka, Tokyo, Japan), followed by size exclusion chromatography (FairJourney Biologics, Porto, Portugal). Isotypes of Motavizumab, a non-human reactive antibody against respiratory syncytium virus, were used as human control IgG (hIgG). The hIgG, the isotype control Fab (derived from Motavizumab), and anti-Dsg3 antibody 4B3 with mouse Fc region (m4B3) were transiently produced in suspension-adapted Chinese hamster ovarian (CHO) K1 cells (ATCC) and purified using MabSelect Sure (by Evitria, Zurich, Switzerland). The isotype control Fab was subjected to additional preparative size exclusion chromatography. Efgartigimod was provided by argenx (Ghent, Belgium). Anti-FcRn Fab (SYNT001) (27), was transiently produced in HEK293-EBNA cells (Thermo Fisher Scientific) and purified twice using CaptureSelect CH1-XL (Life Technologies), followed by gel filtration using a Superdex 200 Increase 16/600 (Sigma-Aldrich) column. The expression and purification were performed by ImmunoPrecise Antibodies (Utrecht, Netherlands). Integrity and size quality controls were performed at the site of production for all antibodies/antibody fragments.

### 2.3 Labeling of antibodies and antibody fragments

Anti-FcRn Fab, control Fab, hAK23-WT, hAK23-IHH, hAK23-DHS (all IgG4), and hIgG4 were labeled with DyLight™ 550 NHS ester (DL550; Cat. 62262; Thermo Fisher Scientific). Efgartigimod and Fc-WT were labeled with DyLight™ 650 NHS ester (DL650; Cat. 62265; Thermo Fisher Scientific). Labeling was performed according to the manufacturer’s protocol, with optimized molar ratios of dye to proteins, and the final degrees of labeling differed by less than 10%. Free dye was removed using 10.000 Da cut-off Vivaspin columns (Cat. No. VS0601; Sartorius, Göttingen, Germany) according to manufacturer’s protocol. The dye removal and ≥ 98% monomeric form were confirmed by high-performance liquid chromatography – size exclusion chromatography (HPLC-SEC; XBridge Protein BEH SEC Column, 200Å, 3.5 µm, 7.8 mm X 300 mm; Cat. 186007640; Waters Corporation, Massachusetts, USA). The free dye for labeled anti-FcRn Fab and control Fab was removed by Zeba™ Dye and Biotin Removal Spin Columns (Cat. A44296; Thermo Fisher Scientific), according to manufacturer’s protocol. All labeled molecules were aliquoted and frozen at -20°C until assayed. Labeled molecules that were used for experiments with living cells were confirmed to be endotoxin free in a LAL-test (ImmunoPrecise Antibodies).

### 2.4 Surface plasmon resonance

Affinity measurements for binding to human FcRn were performed by surface plasmon resonance (SPR), with an IBIS MX96 device (IBIS Technologies, Enschede, Netherlands) and a Continuous Flow Microspotter (Wasatch Microfluidics, Salt Lake City, USA). Biotinylated anti-kappa light chain nanobody (Cat. 7103272500; Thermo Fisher Scientific) was immobilized on a SensEye P Strep (Ssens BV, Enschede, Netherlands) at 30 nM, 10 nM and 3 nM. The Kappa light chain-containing hAK23-F(ab’)2, hAK23-IgG1 and -IgG4 Fc variants were captured by injection of 10 or 20 nM, followed by injection of 2-fold dilution series of soluble human FcRn, starting with 1 µM, in 1× PBS containing 0.075% (v/v) Tween80, at pH 6.0. The sensor was regenerated between the injections of soluble FcRn by injecting 100 mM H_3_PO_4_, pH 1.7. The KD values were calculated using an equilibrium analysis and fitting a Langmuir 1:1 binding model to an R_max_ of 500 RU, using the Scrubber software version 2 (BioLogic, Seyssinet-Pariset, France) and Excel (Microsoft, Washington, USA), as described previously (28).

### 2.5 Flow cytometry

Flow cytometry experiments were carried out on a BD LSRFortessa™ X-20 (BD Biosciences, Ann Arbor, Michigan, USA) with BD FACSDiva Software (BD Biosciences), and the data were analyzed using FlowJo 10.8.1 software (BD Biosciences).

#### 2.5.1 FcRn expression by flow cytometry

Adherent hTert cells were dissociated with trypsin as described previously (26). 1×10^5^ cells were washed twice with ice-cold 1× PBS supplemented with 2% FBS and 2 mM EDTA, and stained at 4°C in the dark for 30 min with 2 nM DL550-labeled anti-FcRn Fab or an irrelevant Fab. These stains were combined with Fixable Viability Stain 520 (Cat. 564407; Pharmingen, Leiden, Netherlands) in 1× PBS supplemented with 2% FBS and 2 mM EDTA at pH 6.0 (surface staining), or permeabilization buffer following fixation at pH 6.0 (total staining; Fixation/Permeabilization kit, Cat. 00-5523; Life Technologies), otherwise following the manufacturer’s instructions. The cells were washed twice with 1× PBS supplemented with 2% FBS and 2 mM EDTA at pH 6.0, and the signals of cell-associated DL550-labeled Fab fragments were measured by flow cytometry.

#### 2.5.2 Intracellular accumulation assay

hTert cells were plated as previously described (26). Briefly, the cells were seeded in 24-well plates (30 000 cells/well) and cultured in complete KGM2 medium (Cat. C20011; PromoCell, Heidelberg, Germany) containing 0.05 mM CaCl_2_ until confluent. Upon confluence, the medium was exchanged to KGM2 with 2 mM CaCl_2_ for 24 h. The cell monolayers were loaded with directly labeled efgartigimod or Fc-WT at 25 µg/ml, or hAK23 IgG4 Fc variants (WT, IHH, DHS), or hIgG (all at 12.5 µg/ml, 1 µg/ml, and 0.1 µg/ml) in KGM2 (2 mM CaCl_2_) for 24 h under culture conditions. All subsequent steps were performed on ice and with pre-cooled buffers. The monolayers were washed with 1× PBS supplemented with 0.05% EDTA (250 µl/well), followed by incubation in 1× PBS supplemented with 0.05% EDTA (500 µl/well) for 20 min. Subsequently, the cells were dissociated with 70 U/ml *Subtilisin A* (Cat. P5459; Sigma-Aldrich), diluted in 1× PBS supplemented with 0.05% EDTA for 40 min, while repeatedly pipetting up and down until obtaining single cells. *Subtilisin A* was neutralized with complete KGM (0.05 mM CaCl_2_), supplemented with 2% FBS (150 µl/well), and the cells were transferred to a 96-well V-bottom plate (Corning, New York, USA).

The cells were then washed with 1× PBS supplemented with 2 mM EDTA and 2% FBS, and stained with Fixable Viability Stain 520 (Cat. 564407; Pharmingen) for 30 min at 4°C, followed by 2 additional washing steps in 1× PBS supplemented with 2 mM EDTA and 2% FBS. Signals of cell-associated, DL650-labeled human IgG1 Fc fragments, DL550-labeled hAK23-IgG4 Fc variants, or IgG were measured by flow cytometry.

#### 2.5.3 Surface Dsg3 binding assay

Cells were plated and dissociated as described for the intracellular accumulation assay. Next, the cells were loaded on ice with directly labeled hAK23-IgG4-WT, or hIgG4 (both at 12.5 µg/ml) in 1× PBS supplemented with 2 mM EDTA and 2% FBS for 15 min. The cells were then washed with 1× PBS supplemented with 2 mM EDTA and 2% FBS, and stained with Fixable Viability Stain 520 (Cat. 564407; Pharmingen) for 30 min at 4°C, followed by 2 additional washing steps in 1× PBS supplemented with 2 mM EDTA and 2% FBS. The signals of cell-associated DL550-labeled hAK23-IgG4-WT or hIgG4 were measured by flow cytometry.

### 2.6 Human FcRn binding ELISA

Nunc MaxiSorp 96-well plates (Thermo Fisher Scientific) were coated overnight at 4°C with 1 μg/ml neutravidin (Cat. PIER31000; Thermo Fisher Scientific) in 1× PBS (PanReac, Cat. A0964; AppliChem, Darmstadt, Germany). Plates were washed with 1× PBS containing 0.05% Tween20 using a 405 Touch Microplate washer (BioTek Instruments, Winooski, USA), and subsequently blocked with 1% (m/v) casein (Cat. C7078; Sigma-Aldrich) in 1×PBS with shaking at 350 rpm at room temperature for 1 h. After washing, biotinylated recombinant human FcRn (1 µg/ml, Cat. ITF01-1000; Immunitrack ApS, Copenhagen, Denmark) was captured on the plates, followed by incubation of a 3-fold dilution series of the indicated antibodies for 1 h at room temperature with shaking at 350 rpm. Binding was assessed with ELISA at both pH 6.0 and pH 7.4, using HRP-conjugated F(ab′)2 directed against human Fc fragment (Cat. ab98595; Abcam, Cambridge, USA). The ELISA assays were developed with 3,3’5,5’-tetramethylbenzidine (TMB, Cat. CL07; Merck, Darmstadt, Germany), and the reactions were stopped with 100 μl 0.5 M H_2_SO_4_. The samples were read using a Tecan Infinite 200 PRO reader (Tecan, Crailsheim, Germany) at 450 nm (620 nm reference).

### 2.7 Anti-Dsg3 ELISA

The MESACUP-2 test Dsg3 enzyme-linked immunosorbent assay (ELISA, Cat. 7886E; MBL, Nagoya, Japan) was used according to the manufacturer’s protocol, with minor modifications. Briefly, a 2-fold dilution series of anti-Dsg3 hAK23 antibody variants (0.01 nM to 0.83 nM) was prepared in the assay diluent. The Fab region of the hAK23 antibodies was detected with 50 ng/ml mouse anti-human kappa light chain, coupled to horseradish peroxidase (HRP) (clone SB81a, Cat. 9230-05; Southern Biotech, Birmingham, USA), diluted in ‘assay diluent’. The substrate was incubated for 60 min.

### 2.8 Monolayer dissociation assay

The monolayer dissociation assays were performed as previously described (25, 26). In short, hTert keratinocytes were seeded in 24-well plates (30 000 cells/well) and grown for at least 3 days to confluence in KGM2 medium with 0.05 mM CaCl_2_. After the cells reached confluence, the medium was exchanged to KGM2 with 2 mM CaCl_2_, and the cells were cultured for a further 24 h to enable stable desmosome assembly. The keratinocyte monolayers were incubated further with various recombinant antibodies: mAK23 (12.5-75 µg/ml), m4B3 (25-100 µg/ml), hAK23 IgG1/IgG4 Fc variants (WT, IHH, DHS; at 0.1-25 µg/ml), human 4B3 (50 µg/ml), or hAK23 F(ab’)2 (8.25-33 µg/ml) for 24 h at 37°C and 5% CO_2_. In all experiments, a treatment with hIgG control antibody was included. In some experiments, a pretreatment with efgartigimod (25 µg/ml; argenx BV, Ghent, Belgium) was additionally applied.

After 24 h treatment with the selected antibody, the cells were washed in Hanks’ Balanced Salt Solution (HBSS, Cat. 14025-050; Gibco, Karlsruhe, Germany) and incubated with 2.5 U/ml Dispase II (Cat. 04942078001; Roche, Mannheim, Germany) solution for about 25 min, until the monolayers were completely detached. The released monolayers were washed again with HBSS and incubated with 3-(4,5-dimethylthiazol-2-yl)-2,5-diphenyltetrazolium bromide (MTT, at 0.25 mg/ml; Sigma-Aldrich) for 15 min. Subsequently, the monolayers were subjected to a defined mechanical stress by pipetting up and down with a 1 ml plastic pipette tip coated with 1% BSA. All samples were included as triplicates, and the experiments were repeated at least three times. The same conditions were applied for all samples. The resulting fragments were imaged and quantified using ImageJ software (29).

### 2.9 Cell lysis, gel electrophoresis and western blot

Western blot analysis of Dsg1 and Dsg3 was performed as described previously (25). In short, confluent hTert keratinocytes were treated with different hAK23-IgG Fc variants for 24 h, and then lysed in a lysis buffer containing 50 mM Tris pH 7.4; 150 mM NaCl; 2 mM EDTA; 1% NP-40, supplemented with a protease inhibitor cocktail (Sigma-Aldrich). Lysates were sonicated to ensure efficient extraction of desmosomal components. Protein concentration of the lysates was assessed using Bradford assay (Bio-Rad, Munich, Germany). Equal amounts of cell extracts (10 µg per sample) were resolved on 10% reducing SDS-PAGE. Precision Plus Protein™ Dual Color standard (Bio-Rad) was used to determine the molecular weight of the selected proteins. After the transfer of proteins onto nitrocellulose membranes, the membranes were blocked for 1 h with 5% (w/v) low fat milk (Carl Roth, Karlsruhe, Germany), followed by overnight incubation at 4°C with primary antibodies: anti-Dsg1, clone B-11 (1:1000, Cat. sc-137164; Santa Cruz Biotechnology, Heidelberg, Germany), anti-Dsg3, clone 5H10 (1:1000, Cat. sc-23912; Santa Cruz Biotechnology), or anti-GAPDH (1:10,000, Cat. ab-8245; Abcam, Cambridge, MA, USA). After extensive washing, the bound primary antibodies were visualized using appropriate HRP-conjugated secondary antibodies (1:10 000; Dako, Glostrup, Denmark). The signals were detected by enhanced chemiluminescence assay on a roentgen film. To quantify the signals, densitometric analysis of scanned films was performed using the ImageJ software (29). Intensity of the signal was normalized to GAPDH.

### 2.10 Statistical analysis

Statistical analysis and data presentation were done using GraphPad Prism 5.0 or 8.0 (GraphPad Software Inc., San Diego, CA, USA) unless otherwise indicated. Depending on the data set, a Welch ANOVA (Dunnett’s T3 multiple-comparison test) or one-way ANOVA (Dunnett’s multiple-comparison test) were applied. In some cases, two-way ANOVA with Bonferroni’s post-test was performed, as specified in the figure legends. Statistically significant differences were indicated by asterisks *p ≤ 0.05, **p ≤ 0.01, ***p ≤ 0.001, ****p ≤ 0.0001.

## 3 Results

**Figure 1:**
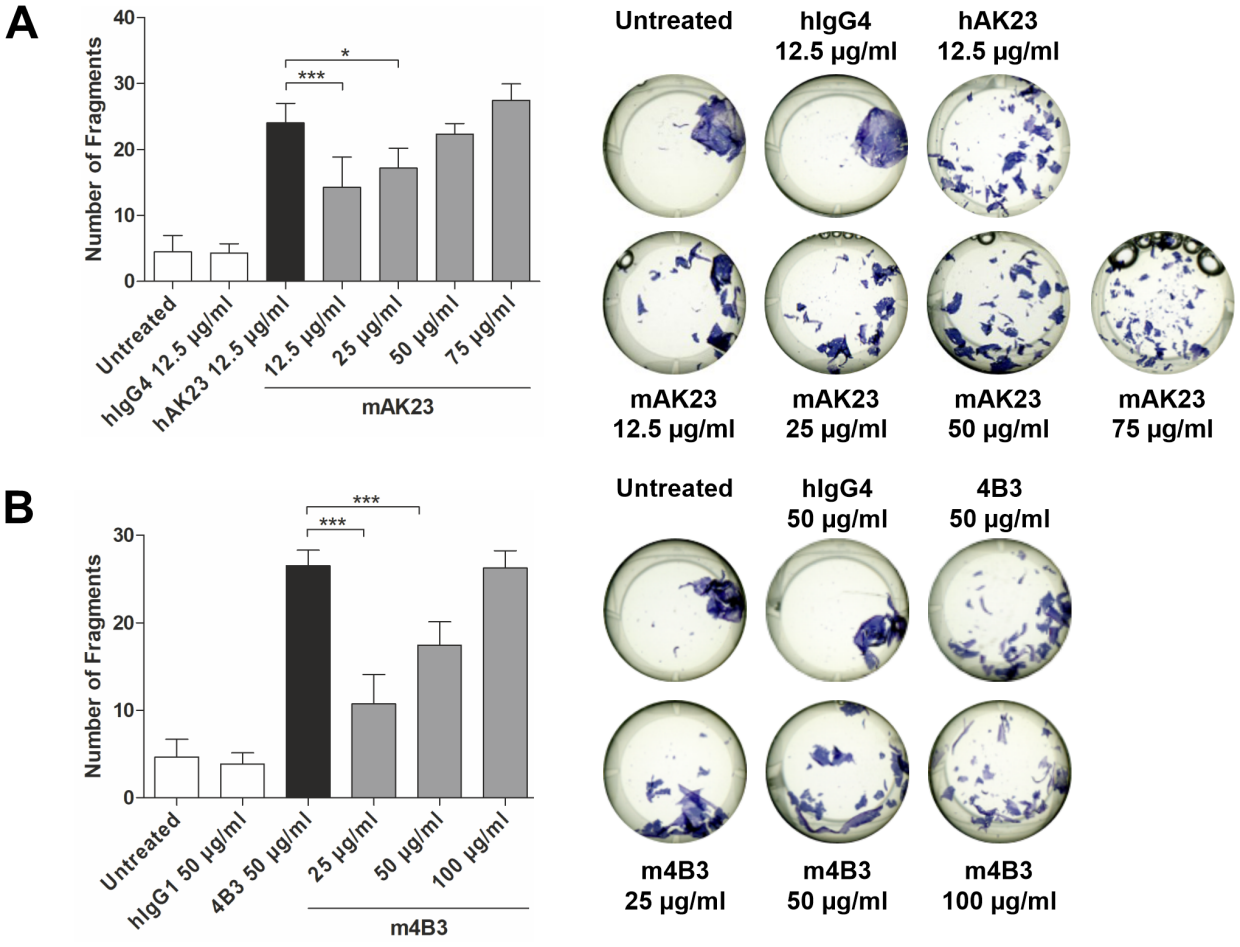
Recombinant mouse anti-Dsg3 antibodies AK23 and 4B3 are less pathogenic in the monolayer dissociation assay than chimeric antibody variants that contain a human Fc region. The hTert cells were cultured in KGM2 with 0.05 mM CaCl_2_ until confluent, after which the CaCl_2_ concentration was increased to 2 mM for another 24 h. Thereafter, dose-dependent effect of a 24 h treatment with antibodies containing mouse Fc region **(A)** mAK23 (12.5-75 µg/ml) or **(B)** m4B3 (25-100 µg/ml) on the monolayer integrity was analyzed using the monolayer dissociation assay. Antibody variants with human Fc region **(A)** hAK23 (12.5 µg/ml) or **(B)** 4B3 (50 µg/ml) were used as a positive controls. Isotype-matched, non-human reactive recombinant antibody hIgG4 (12.5 µg/ml) or hIgG1 (50 µg/ml) and untreated cells were included as controls. The number of fragments was quantified using the ImageJ software. One representative well for each treatment is shown. The error bars represent the SD of the mean values obtained from at least four independent experiments, each of which was performed in triplicates. Statistical analysis was done using one-way analysis of variance (ANOVA) with Dunnett’s post-test. Statistically significant differences to hAK23 or human 4B3 are indicated by asterisks. * = *p* ≤ 0.05; *** = *p* ≤ 0.001.

We have earlier shown that blockade of anti-Dsg3 antibody binding to FcRn in keratinocytes by efgartigimod results in lower pathogenicity of the antibodies in a monolayer dissociation assay (25). These findings pointed to a novel role for FcRn in the pathogenesis of autoimmune diseases like pemphigus and prompted us to study the consequences of FcRn engagement in this context. To understand the role of FcRn in autoantibody pathogenicity, we used recombinant AK23 and 4B3 (25, 30, 31) antibodies in mouse (mAK23 and m4B3) and human (hAK23 and 4B3) Fc formats that are identical in their antigen-binding Fab regions. Since mouse IgG antibodies exhibit negligible binding to human FcRn (32), we compared the pathogenicity of recombinant anti-Dsg3 antibodies with a human IgG Fc region (25) with the respective recombinant antibodies with a mouse Fc region. As controls, non-human reactive recombinant antibodies with a human Fc region (hIgG1 or hIgG4) were used. In our monolayer dissociation assay with human hTert keratinocytes, at least a 4-fold higher concentration of mAK23 was required to induce the same number of fragment as hAK23 (IgG4; 50 µg/ml vs. 12.5 µg/ml; **Figure 1A**). In the case of the recombinant anti-Dsg3 antibody 4B3, about double the amount of the respective m4B3 antibody (IgG1; 50 µg/ml vs. 100 µg/ml) was required for an equal level of fragmentation (**Figure 1B**). Since the recombinant antibody pairs exhibit identical antigen-binding regions, their Fc regions that mediate the interaction with FcRn appeared to affect their pathogenicity.

**Figure 2:**
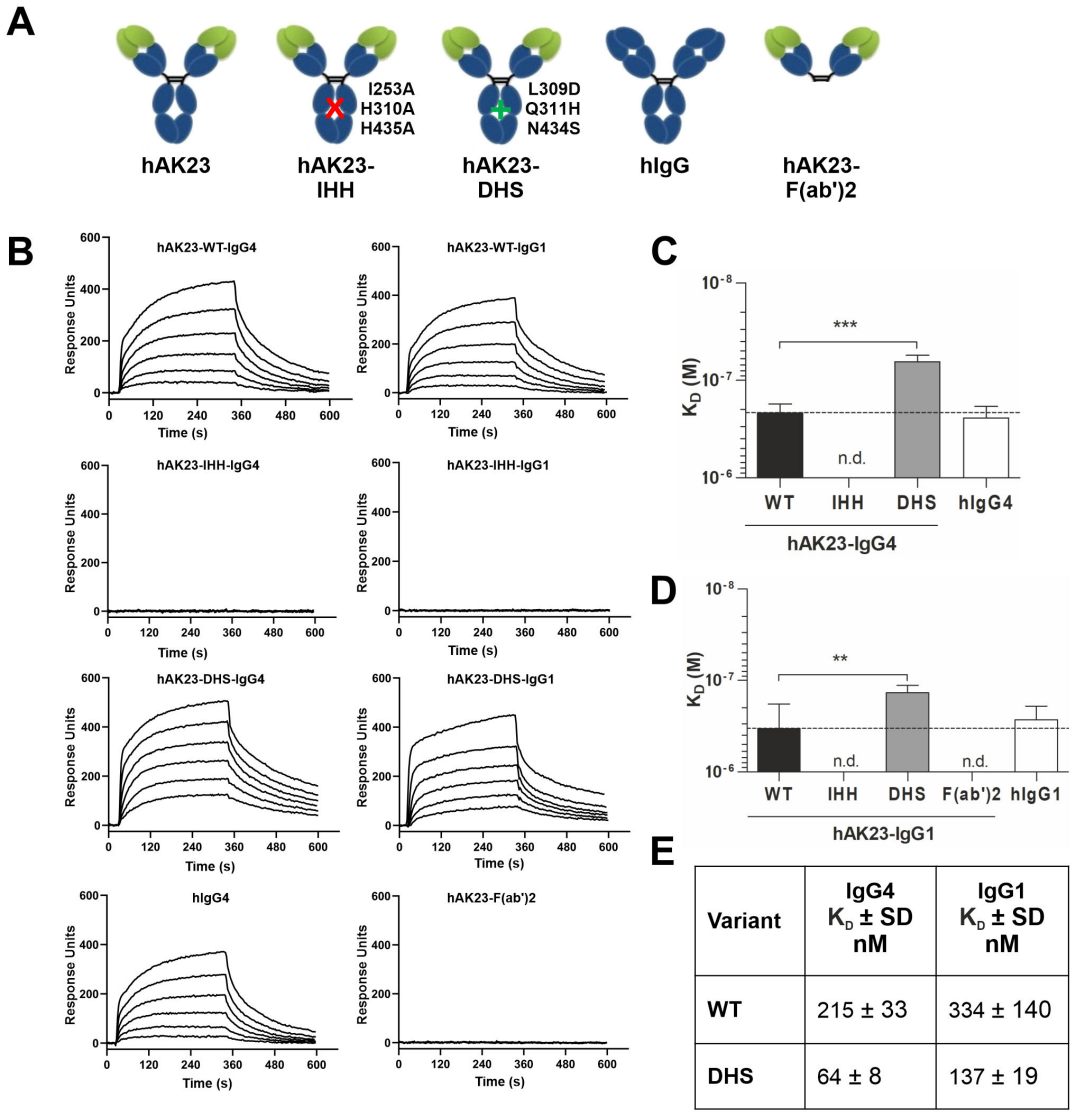
Human AK23 IgG Fc variants and F(ab’)2 show desired binding properties to soluble human FcRn by SPR at pH 6.0. **(A)** Schematic presentation of the recombinant anti-Dsg3 monoclonal AK23 antibody variants. **(B)** Representative sensorgrams of soluble human FcRn binding to captured hAK23 IgG Fc variants or hAK23-F(ab’)2, obtained from SPR experiments at pH 6.0. A biotinylated anti-kappa nanobody was immobilized on a streptavidin sensor at 30 nM, 10 nM and 3 nM, and 10 or 20 nM of the kappa light chain-containing hAK23-IgG Fc variants, hAK23-F(ab’)2, or hIgG as isotype control were captured, followed by injection of a 2-fold dilution series of soluble human FcRn (starting at 500 nM). The sensor surface was regenerated between the injections. **(C, D)** Comparison of the K_D_ values of the SPR experiments shown in **(B)**. The K_D_ values were obtained using 1:1 equilibrium analysis and fitting a Langmuir binding model. n.d., not determinable. Error bars indicate standard deviations. Statistical analysis was performed by Welch ANOVA (IgG1) or ANOVA test (IgG4), with pairwise comparisons to the corresponding hAK23-WT. The *p* values were corrected by applying Dunnett’s T3 (IgG1) or Dunnett’s multiplicity correction (IgG4) to control overall type I error at 5%. Statistical differences are indicated with asterisks: ** = *p <*0.01; *** = *p <*0.001. **(E)** K_D_ values ± SD (nM) for the WT and DHS variants, derived from data shown in **(C)** and **(D)**.

To study this further, we designed hAK23 Fc variants that differ in their binding to FcRn due to targeted amino acid changes in the Fc region (**Figure 2A**). Importantly, all variants showed highly similar antigen binding, demonstrating that the Fc modifications do not affect the antigen binding properties (**Supplementary Figures S1A, B**). The DHS variant exhibits an enhanced binding to FcRn at pH 6.0, compared to WT IgG, whereas the IHH substitutions in the Fc region abrogate FcRn interaction (10, 17). We determined the FcRn binding affinities of these variants using SPR (**Figure 2B**). While the hAK23 and control hIgG with WT Fc regions showed highly similar dissociation constants (K_D_), the hAK23-DHS variants exhibited a significantly stronger binding to FcRn, and the hAK23-IHH variant and hAK23 F(ab’)2 fragment showed no binding at pH 6.0 (**Figures 2B–D**). The dissociation constants of the WT and DHS variants with IgG1 or IgG4 Fc regions are shown in **Figure 2E**.

**Figure 3:**
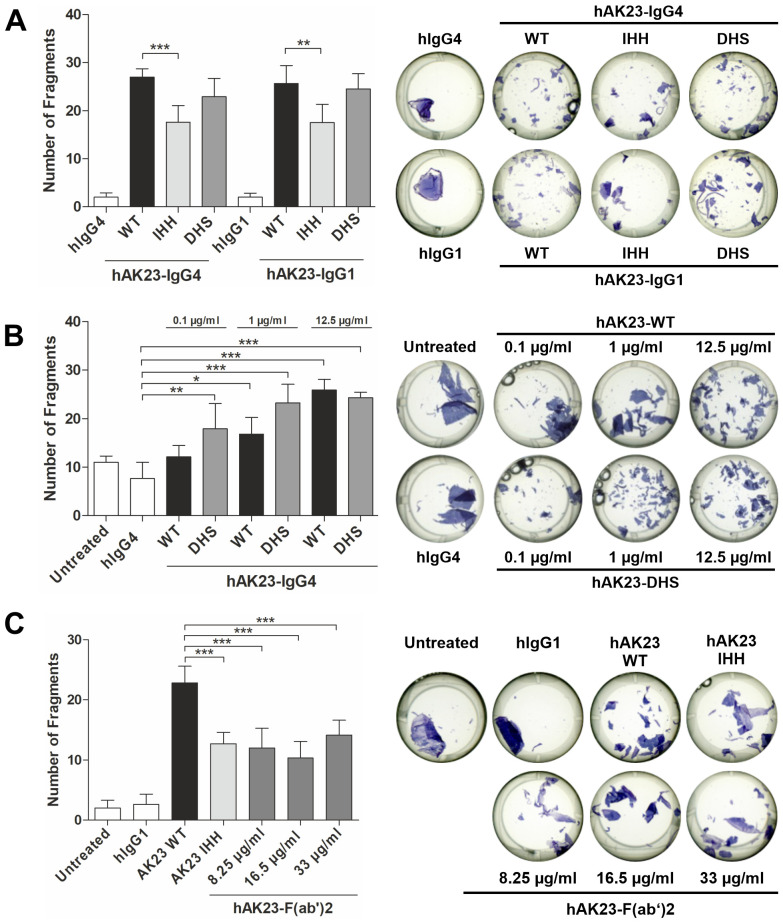
Degree of pathogenicity of hAK23 antibodies in the monolayer dissociation assay depends on their FcRn binding profile, but not on the IgG subclass. The cells were cultured as described in **Figure 1**, and then treated for 24 h with: **(A)** the Fc variants WT, IHH, or DHS of hAK23-IgG4 or -IgG1 (12.5 µg/ml); **(B)** hAK23-IgG4-WT or -DHS variants (0.1 µg/ml - 12.5 µg/ml); or **(C)** hAK23-IgG1-F(ab’)2 (8.25 µg/ml - 33 µg/ml). As controls, mock incubation (untreated), hIgG4 or hIgG1 (12.5 µg/ml) were applied. The monolayer dissociation assay was performed as described in **Figure 1**. The error bars represent the SD of the mean values obtained from at least four independent experiments, each of which was performed in triplicate. Statistical analysis was done using one-way ANOVA with Dunnett’s post-test. Statistically significant differences compared to the appropriate positive or negative control (as indicated in the graphs) are indicated with asterisks. * = *p* ≤ 0.05; ** = *p* ≤ 0.01; *** = *p* ≤ 0.001.

We next tested if the FcRn binding affinity of the hAK23 antibody variants (IgG1 and IgG4) affects their pathogenicity in the monolayer dissociation assay (**Figure 3**). The hAK23-IHH variant induced significantly less fragments than the hAK23-WT, whereas there was no significant difference between the hAK23-WT and -DHS variants at 12.5 µg/ml (**Figure 3A**). Since no differences between IgG1 and IgG4 antibody variants were observed, the following experiments were performed with IgG4 variants only. At lower concentrations (0.1 and 1 µg/ml), the hAK23-DHS variant (IgG4) produced a moderately higher fractionation compared to hAK23-WT (**Figure 3B**). At 0.1 µg/ml, only the hAK23-DHS variant exhibited a significant difference to the control (hIgG4), whereas the hAK23-WT did not. At 1 µg/ml, hAK23-DHS more significantly differed from the control than the WT did. A recombinant hAK23 F(ab’)2 fragment exhibited a pathogenic effect about equal to the hAK23-IHH variant, but without any clear dose dependency (**Figure 3C**). These data suggest that FcRn binding of the anti-Dsg3 antibodies is important for their pathogenic effect in the monolayer dissociation assay.

**Figure 4:**
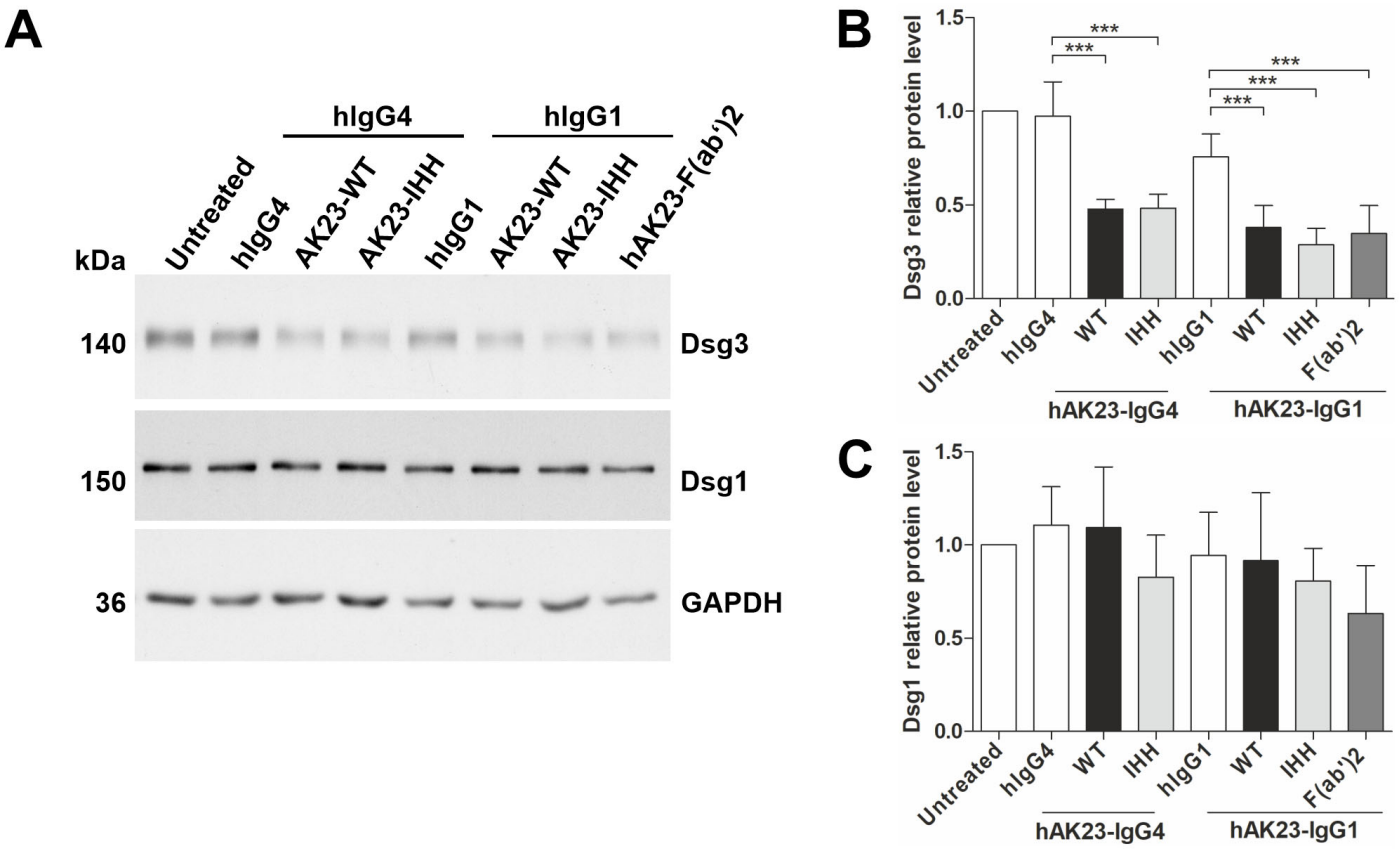
Antibody-induced Dsg3 degradation is not dependent on the FcRn binding profile of the antibody. The hTert cells were grown as described in **Figure 1** and treated for 24 h with the Fc variants of hAK23-IgG4 or -IgG1 (12.5 µg/ml) or hAK23-F(ab’)2 (8.25 µg/ml). Mock incubation (untreated), hIgG1 or IgG4 (12.5 µg/ml) were included as controls. **(A)** The level of Dsg3 and Dsg1 in cell lysates was assessed by Western blot. GAPDH served as a loading control. Dsg3 **(B)** and Dsg1 **(C)** were quantified by densitometry using ImageJ software, normalized against GAPDH, and expressed as relative values compared to the respective hIgG control. The error bars represent the SD of data from four independent experiments. Statistical analysis was done using one-way ANOVA with Dunnett´s post-test. Statistically significant differences are indicated by *** = *p* ≤ 0.001.

According to our earlier findings, anti-Dsg3 antibodies can induce Dsg3 degradation in keratinocytes (25), but the contribution of the Fc-FcRn interaction to Dsg3 degradation has not been directly assessed. Loss of Dsg3 expression is not dependent on the FcRn binding profile of the anti-Dsg3 antibody variants, as both the IHH variant and the hAK23 F(ab’)2 fragment induced Dsg3 degradation as efficiently as WT hAK23, irrespective of the IgG class (IgG1 vs. IgG4, **Figures 4A, B**). However, Dsg1 protein level was not significantly changed by any of the recombinant antibody variants (**Figures 4A, C**). These data demonstrate that the induction of Dsg3 degradation by anti-Dsg3 antibodies does not depend on FcRn binding propensity of the antibodies.

We have earlier demonstrated that FcRn is expressed in human hTert keratinocytes (25), but the cellular localization of FcRn in these cells has so far not been studied. Flow cytometry with a DL550-labeled anti-FcRn Fab and isotype control Fab fragment was used to assess FcRn localization in hTert keratinocytes. As a control, THP-1 cells that express higher amounts of FcRn were used (33). In hTert keratinocytes, most of the FcRn resides in intracellular compartments, whereas in THP-1 cells, a substantial fraction is also present at the cell surface (**Supplementary Figure S2A**).

**Figure 5:**
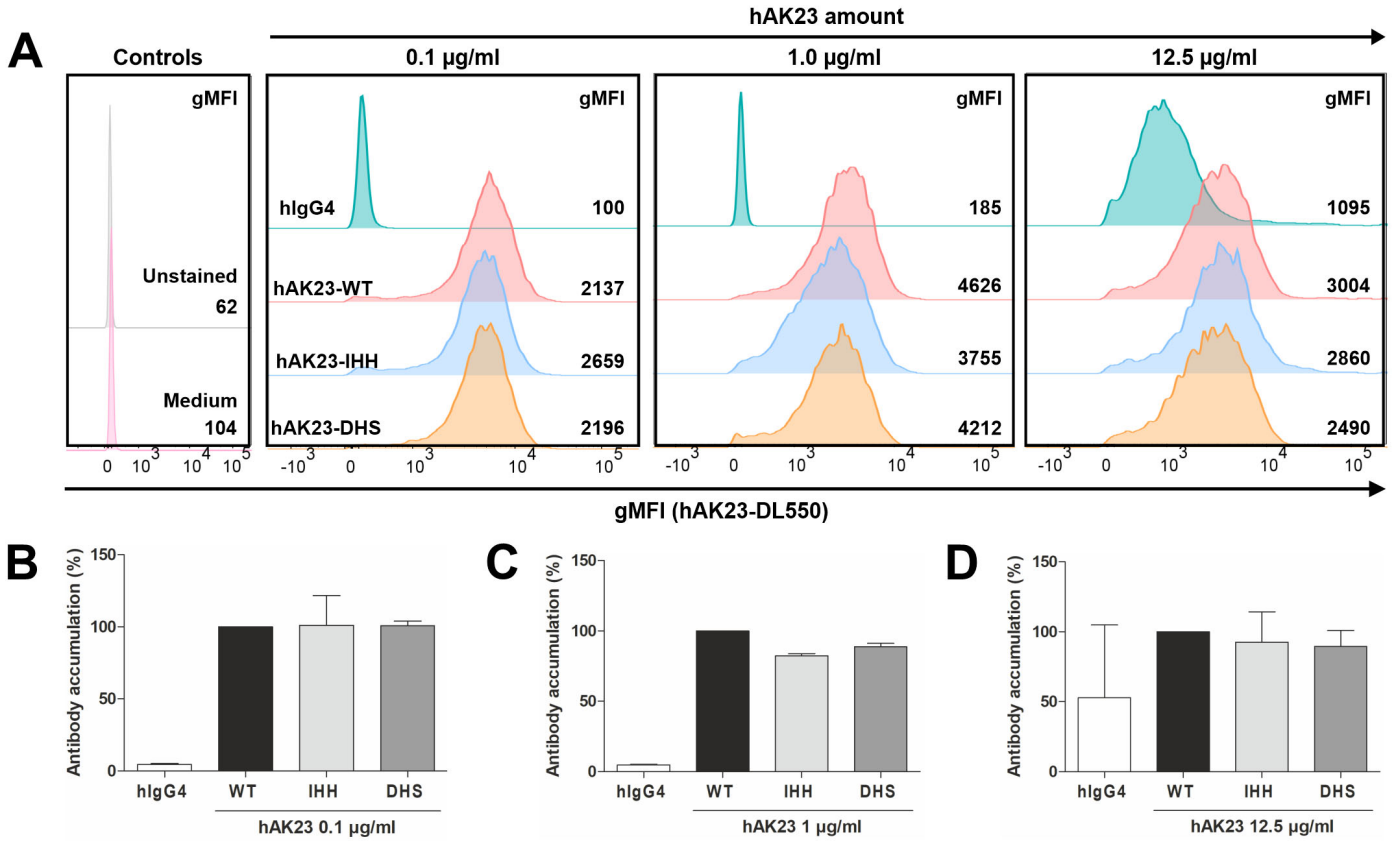
Anti-Dsg3 antibodies enter keratinocytes in a target-mediated manner that is not influenced by FcRn binding. **(A)** Representative flow cytometry histograms of an intracellular accumulation experiment in hTert cells for comparison of signals of DL550-labeled hAK23-IgG4 Fc variants (WT, DHS, IHH) and hIgG. Numbers indicate averaged gMFI values of two technical replicates (one replicate for unstained control). The cells were loaded for 24 h with 0.1, 1.0 or 12.5 µg/ml hAK23-IgG4 Fc variants, washed extensively, and analyzed by flow cytometry. **(B-D)** Relative intracellular accumulation of the antibody variants normalized to hAK23-IgG4-WT for each concentration. The gMFI values for each concentration were derived from at least three independent experiments and are thus not comparable between the experiments. At least 3200 events in the live gate were acquired. The data represent the mean of the averaged, normalized values from at least three independent experiments and are given as relative accumulation of the respective antibody (% WT). Error bars indicate standard deviations.

To further study the recombinant anti-Dsg3 antibody variants in hTert cells, the variants were labeled with DL550, and the binding of the labeled vs. unlabeled variants to FcRn was verified by ELISA (**Supplementary Figure S2B**). All labeled variants showed the expected binding profile to FcRn at pH 6.0, with the DHS variant binding more efficiently than the other ones. The binding profiles of the labeled vs. unlabeled variants were nearly identical, demonstrating that the labeling did not affect binding to FcRn (**Supplementary Figure S2B**). No binding was observed at pH 7.4, consistent with the pH-dependent binding mode of human IgG antibodies to FcRn (**Supplementary Figure S2B**). To study if the intracellular accumulation of the anti-Dsg3 antibody variants in hTert keratinocytes is dependent on FcRn, flow cytometry was applied. The FcRn binding profile did not affect the accumulation of the anti-Dsg3 hAK23-WT, -IHH and -DHS variants, but at the highest concentration used (12.5 µg/ml), some uptake of the isotype control, most likely by fluid-phase endocytosis, was observed in some experiments (**Figures 5A–D**).

Upon low Ca^2+^ (0.05 mM), Dsg3 expression in hTert cells is low, and Dsg3 is intracellularly localized, whereas at 2 mM Ca^2+^, Dsg3 is abundantly present at the plasma membrane (26). The cells were incubated at 0.05 mM (no surface Dsg3) or 2 mM Ca^2+^ (Dsg3 at cell surface) with 12.5 µg/ml DL550-labeled hAK23-IgG4 or control IgG4 for 24 hours at 37°C, and then transferred to 4°C. The monolayer was dissociated, and the surface-associated anti-Dsg3 was removed with cold-active protease *Subtilisin A* (schematic of the method shown in **Supplementary Figures S3A, B**). The intracellularly accumulated antibodies were measured by flow cytometry. The presence of Dsg3 at the cell surface was required for the uptake of anti-Dsg3 antibodies (**Supplementary Figure S3C**). The efficiency of the removal of surface Dsg3 was controlled under conditions where the dissociation was done prior to treatment with anti-Dsg3 antibodies (**Supplementary Figure S4**). Together, these data show that the uptake of anti-Dsg3 antibodies into keratinocytes requires surface Dsg3, whereas FcRn is not substantially involved.

Efgartigimod, a modified IgG1 Fc fragment that shows enhanced binding to FcRn due to the ABDEG substitutions in its Fc region (22), intracellularly accumulated in hTert keratinocytes significantly more than the WT Fc fragment (**Supplementary Figures S5A, B**). The binding of efgartigimod to FcRn was not altered by the fluorescent labeling, as demonstrated by ELISA (**Supplementary Figures S5C, D**). These data indicate that the intracellular accumulation of Fc fragments that lack Fab regions can be enhanced by increasing their affinity to FcRn at pH 7.4, as described previously (10, 22).

**Figure 6:**
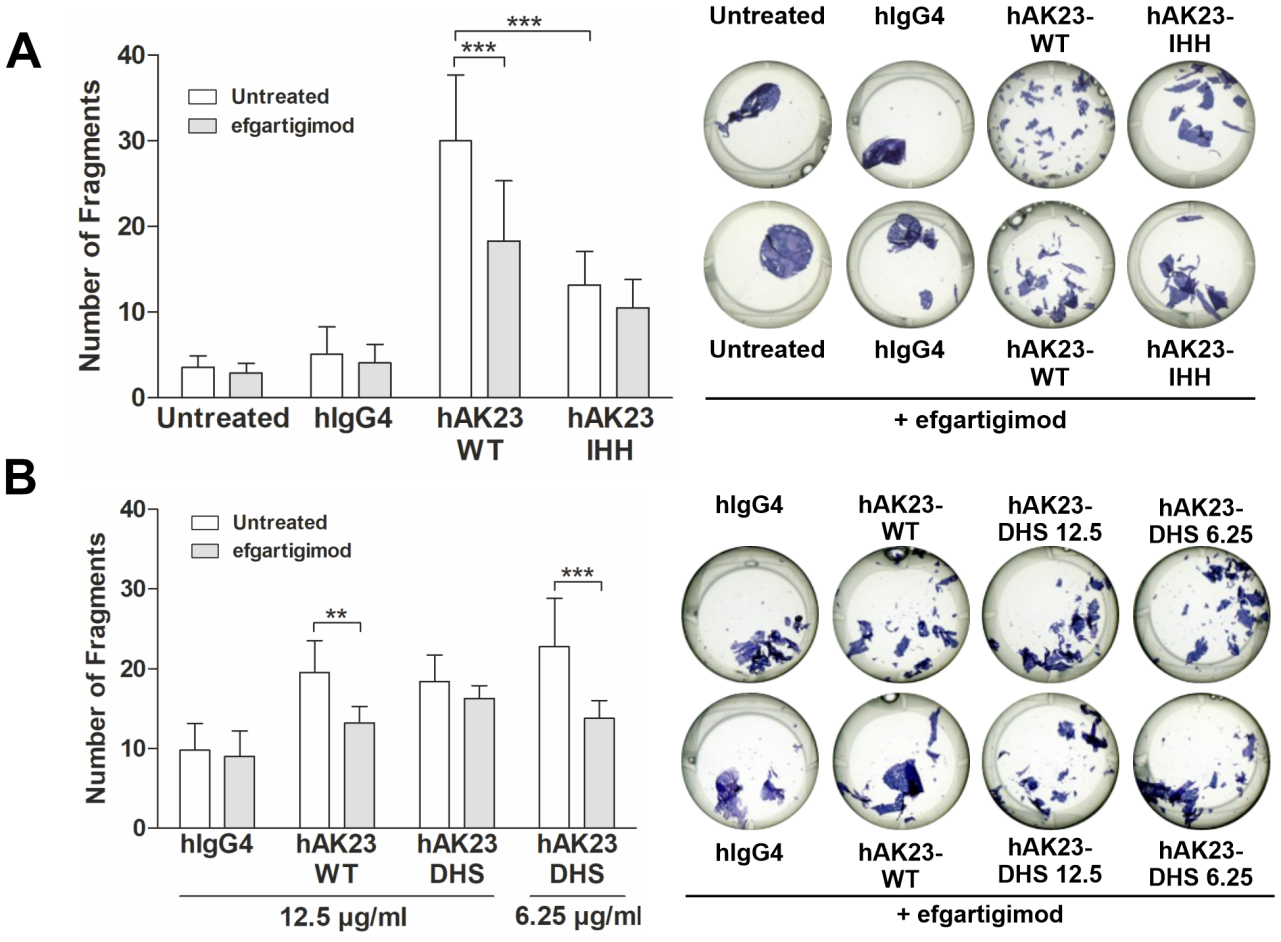
Efgartigimod treatment protects keratinocyte monolayers against dissociation induced by hAK23 WT and DHS but not by IHH variants. The hTert cells were cultured as described in **Figure 1**. The cells were first treated with efgartigimod (25 µg/ml) for 25 min or left untreated, and then incubated with the antibody variants for 24 h. Mock incubation (untreated) and hIgG4, (12.5 µg/ml) were used as negative controls. The integrity of the keratinocyte monolayer was analyzed by monolayer dissociation assay. **(A)** hAK23-WT and IHH (IgG4, 12.5 µg/ml); **(B)** WT and DHS (12.5 or 6.25 µg/ml) were then applied. The number of fragments was quantified using the ImageJ software. Representative images for each treatment are shown. The error bars represent the SD of the mean values obtained from at least four independent experiments, each of which was performed in triplicate. Statistical analysis was done with two-way ANOVA with Bonferroni’s post-test. Statistically significant differences are indicated by asterisks. ** = *p* ≤ 0.01,*** = *p* ≤ 0.001.

FcRn blockade by efgartigimod reduces the number of fragments induced by anti-Dsg3 antibodies in the keratinocyte monolayer dissociation assay (25). Efgartigimod significantly (by about 40%) reduced the number of fragments induced by hAK23-WT (IgG4), but not by hAK23-IHH, which exhibited a lower fragmentation than the hAK23-WT already in the absence of efgartigimod (**Figure 6A**). Efgartigimod also showed no effect on the fragmentation induced by 12.5 µg/ml hAK23-DHS, whereas at 6.25 µg/ml, a significant inhibition by efgartigimod (about 40% reduction) was observed (**Figure 6B**). This may be due to the enhanced binding affinity of the DHS variant to FcRn at acidic pH, and thus more efficient competition with efgartigimod for the binding to FcRn in endosomes. Taken together, these data further demonstrate that FcRn binding affinity is important for the full pathogenicity of anti-Dsg3 antibodies in keratinocytes, but a basal level of fragmentation can take place even in the absence of FcRn binding.

## 4 Discussion

FcRn was originally suggested by Brambell to function as a receptor for the transport of IgG antibodies from the mother to the fetus (1), but it is now considered to be involved in a plethora of further cellular processes (2, 3). Keen interest in FcRn biology has been boosted by the findings demonstrating that blocking IgG binding to FcRn results in beneficial therapeutic effects in IgG-mediated autoimmune diseases, such as MG, CIDP and ITP (19–21). In such diseases, the half-life of the disease-relevant IgG autoantibodies is considerably shortened due to impaired recycling and enhanced degradation upon FcRn blockade, which results in amelioration of the disease symptoms.

We previously demonstrated that in cultured human keratinocytes, blockade of FcRn with efgartigimod, a human Fc fragment with enhanced FcRn binding, was protective against keratinocyte monolayer dissociation caused by pathogenic anti-Dsg3 monoclonal antibodies mAK23 and 4B3 (25, 30, 31). Already in this study, we noted that the hybridoma-derived mouse AK23-IgG that only poorly binds to human FcRn, typically of mouse IgG (32), was less pathogenic (i.e. caused fewer fragments in our quantitative monolayer dissociation assay) than the corresponding chimeric antibody with a human Fc region, despite identical antigen binding regions. This raised the question if FcRn binding was modulating the pathogenic effect of the anti-Dsg3 antibodies. In the present study, we demonstrated that recombinant anti-Dsg3 mAK23 and m4B3 IgG4 monoclonal antibodies with a murine Fc region were less pathogenic, inducing about 40% less fragments than their counterparts with a human Fc part (hAK23 and 4B3). Therefore, FcRn binding affinity indeed appears to enhance the pathogenicity of anti-Dsg3 antibodies, but FcRn binding is not essential for the pathogenic effect in this assay.

We further addressed this question by generating recombinant monoclonal antibodies that carry amino acid substitutions in their Fc region (human) that differentially modulate their binding affinity to FcRn. The recombinant hAK23 IHH variant that does not bind to FcRn, and the hAK23 F(ab’)2 exhibited significantly lower monolayer fragmentation (about 40% less fragments) than the hAK23-WT or -DHS (with enhanced FcRn binding affinity). While the DHS variant did not significantly differ from the WT at the standard concentration used (12.5 µg/ml), it induced a more pronounced fragmentation at low concentrations (0.1 – 1 µg/ml), suggesting a mildly increased pathogenic effect, consistent with its higher affinity to FcRn. However, loss of FcRn binding does not completely abrogate the pathogenicity of anti-Dsg3 antibodies, but FcRn interaction is important for the full pathogenic effect in our quantitative assay, even though both FcRn-mediated and FcRn-independent effects appear to play a role.

In PV, IgG4 subclass autoantibodies are the predominant form during an active disease phase (34–36). We did not detect any significant differences between recombinant hAK23-IgG1 vs. -IgG4 in the pathogenicity or FcRn binding. However, our recombinant hAK23-IgG4 contains the Ser228Pro substitution that prevents the Fab arm exchange, which is a natural feature of endogenous human IgG4 antibodies and results in bi-specific (two different antigen binding specificities) IgG4 molecules (37, 38). This may affect e.g. the clustering of the target antigens of such bi-specific IgG4 molecules in PV, resulting in different acantholysis and signaling responses. This in turn may modulate the pathogenic outcome of the IgG4 antibodies, but the mono- and bi-specificity of the IgG4 autoantibodies in PV has so far not been addressed in detail. However, as the Fc regions and thus the binding to FcRn should not be affected by the potential bi-specificity of IgG4, our mono-specific recombinant IgG4 antibodies should also correctly reflect the role of FcRn in our cell system.

Different from IgG1 and IgG4 with high FcRn affinity, IgG3 only poorly binds to FcRn, resulting in lower half-life of these molecules that are usually found in early phases of an immune reaction, immediately following IgM. Natural variants of the Fc region in IgG3, such as the Arg435His substitution, result in a higher FcRn binding affinity and an increased half-life of IgG3 (39). Importantly, Arg435His variant is associated with a higher risk of PV, but not of bullous pemphigoid, in certain populations (40). Thus, it has been postulated that in the early phases of PV, anti-Dsg3 autoantibodies of the IgG3 class are present, and their FcRn-mediated stabilization due to the Arg435His variant might promote the disease (40). Therefore, an early intervention with FcRn blocking agents in PV patients that exhibit the Arg435His variant might be beneficial to prevent further disease progression.

Previous studies have suggested that FcRn-dependent mechanisms and the Fc part of anti-Dsg3 antibodies would not be required for blister formation in the passive transfer neonatal mouse model that is frequently used to assess the *in vivo* pathogenicity of anti-Dsg antibodies (41–43). Even though this may appear to be discrepant with our present findings, it has to be kept in mind that the passive transfer blistering model is not quantitative, and is thus not capable of differentiating between full vs. reduced pathogenicity. Therefore, the contribution of FcRn and the Fc part of IgG to the pathogenicity of anti-Dsg3 antibodies may remain undetected in this system. In contrast, our monolayer fragmentation assay can detect even subtle quantitative differences between different antibodies and is thus more suitable for studies comparing the degree of pathogenicity. On the other hand, the passive transfer method is an *in vivo* assay, whereas our monolayer fragmentation assay does not fully recapitulate the multilayer epidermis in which the antibodies need to gain access to their antigens by passing several cell layers.

It is possible that in the epidermis, FcRn may mediate the transfer of pathogenic PV antibodies through the cell layers, so that they can access desmosomes beyond the basal layer of the epidermis. Therefore, one possible mechanism for efgartigimod action in the treatment of pemphigus could be blockade of antibody access to their antigens in the epidermis, as previously suggested by us (25). This would be consistent with the function of FcRn in IgG transcytosis in other epithelial tissues, as shown in the intestine and placenta (3). It has been suggested recently that in bullous pemphigoid (BP), another autoimmune blistering disease of the skin, FcRn blockage might lead to enhanced local degradation of autoantibodies (44). These possibilities should be directly addressed in future studies on PV and BP using suitable 3-dimensional cell systems or tissue-specific FcRn knockouts, as suggested by Bao et al. (44).

FcRn also mediates the intracellular trafficking of immune complexes (ICs) consisting of the IgG molecule and their respective antigens [for details, see the review by Pyzik et al. (2)]. Small IgG-ICs are apparently sorted by FcRn into tubules of recycling endosomes, and thus transported back to the cell surface, similarly to IgG molecules. In contrast, multimeric IgG-ICs fail to be recycled and are preferentially targeted to lysosomal compartments (45). Upon FcRn blockade, not only serum IgGs are reduced, but also IgG-ICs, demonstrating the role of FcRn in these processes (27, 46, 47). The exact mechanisms of differential sorting of small vs. large ICs by FcRn are currently not understood in detail, and most of the studies addressing the role of FcRn in IgG-IC trafficking have been performed with soluble IgG-ICs. However, in PV, the Dsg-IgG-ICs are expected to be, at least in part, membrane bound. According to our data, Dsg3 degradation upon anti-Dsg3 antibody treatment and the destabilizing effect of anti-Dsg3-IgG on the keratinocyte monolayer integrity appear not to be directly coupled, and FcRn blockade does not prevent or reduce Dsg3 degradation (25). Furthermore, anti-Dsg3-IgG not capable of binding to FcRn also causes Dsg3 degradation, as shown in the present study. These data show that the stabilizing effect of FcRn blockade on the keratinocyte monolayer occurs through a mechanism different from protection of Dsg3-ICs from degradation, and FcRn-mediated sorting is not required for Dsg3 degradation.

In this study, we have for the first time demonstrated that binding of anti-Dsg3 antibodies to FcRn enhances their pathogenic effect. Our findings may be relevant for the therapy of IgG-mediated autoimmune diseases such as PV, but may also be of importance for further autoimmune diseases that are based on FcRn-binding IgG, such as Graves disease, ITP and MG. So far, the role of FcRn in such diseases has been suggested to be mainly associated with the recycling and prolongation of the half-life of IgG. However, our data demonstrate that FcRn blockade with efgartigimod also directly reduces the pathogenic effect of autoantibodies in keratinocytes, and this effect cannot be attributed to the reduced IgG half-life. Therefore, the role of FcRn in autoimmune diseases is likely to be more versatile than merely IgG recycling. Further studies on the detailed molecular mechanisms of the FcRn-mediated enhancement of pathogenicity of autoantibodies, and the stabilizing effect of FcRn blockade with compounds such as efgartigimod are thus required. One possible mechanism of this enhancement is related to the various signaling pathways that play a major role in mediating the pathogenic effects in PV (48).

## Data Availability

The raw data supporting the conclusions of this article will be made available by the authors, without undue reservation.
