# Supplementary material for: Binding to the neonatal Fc receptor enhances the pathogenicity of anti-desmoglein-3 antibodies in keratinocytes
Source: Front Immunol. 2024 Oct 10;15:1473637. doi: 10.3389/fimmu.2024.1473637 (PMC11499148; doi:10.3389/fimmu.2024.1473637)
Supplement: Supplementary file 1 [file DataSheet1.pdf]

frontiers

Supplementary Material for

Binding to the Neonatal Fc receptor enhances the pathogenicity of anti-desmoglein-3 antibodies in keratinocytes

Zakrzewicz et al.

Corresponding author: Ritva Tikkanen

Ritva.Tikkanen@biochemie.med.uni-giessen.de

The file contains Supplementary Figures S1 – S5

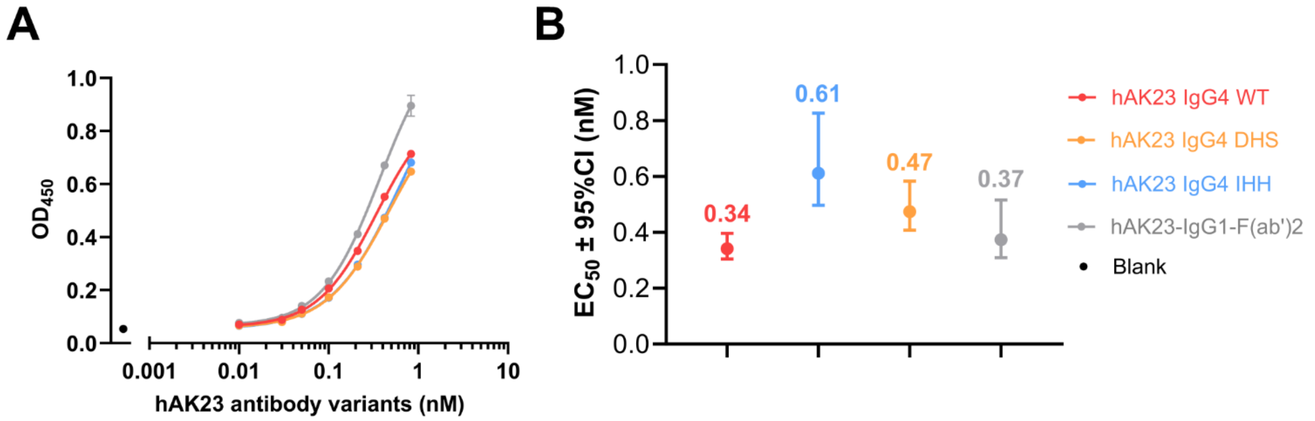

Supplementary Figure S1. Recombinant hAK23 IgG Fc variants and F(ab')2 show similar apparent affinity (EC_{50}) to human Dsg3 by ELISA at pH 7.4. (A) Dose-response curves of hAK23-IgG4-WT, DHS, IHH, and hAK23-IgG1-F(ab')2 binding to human Dsg3, obtained by ELISA at pH 7.4. Increasing amounts of hAK23-IgG4 Fc variants or IgG1-F(ab')2 (0.01 nM to 0.83 nM) were captured on immobilized recombinant human Dsg3, followed by detection of the bound hAK23 (Fab region) by an HRP-conjugated anti-human kappa light chain antibody. Data represents the averaged OD_{450} values of two technical replicates from each experiment. Error bars indicate standard deviations. (B) EC_{50} values (±95% CI) for binding of hAK23-IgG4 Fc variants and hAK23-IgG1-F(ab')2 to human Dsg3, calculated from data presented in (A). Error bars indicate standard deviations. EC_{50}: half-maximal effective concentration, CI: confidence interval.

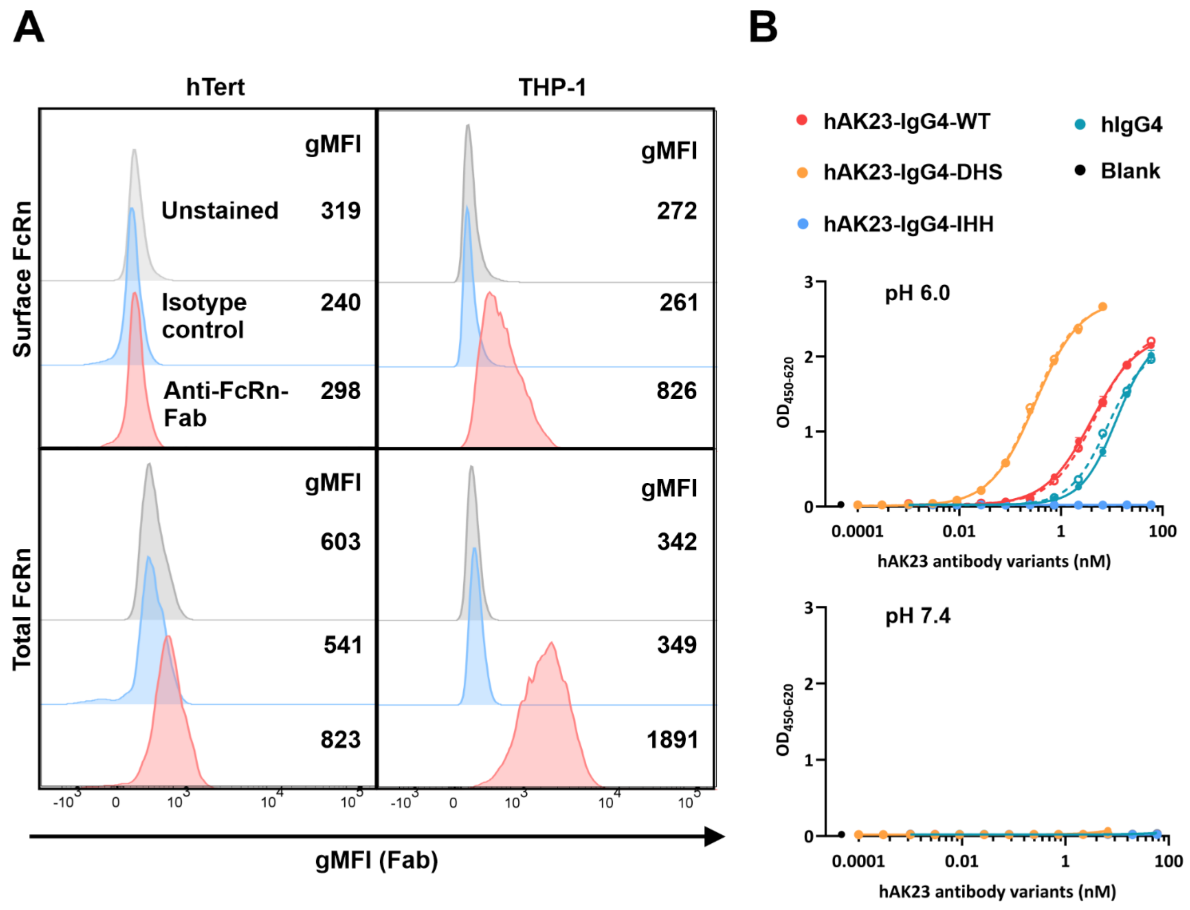

Supplementary Figure S2. Keratinocytes intracellularly express FcRn, and the binding of hAK23-IgG4 Fc variants to human FcRn is not impacted by fluorescent labeling. (A) Representative flow cytometry histograms of human FcRn signal in comparison to isotype and unstained control in hTert (left), and THP-1 cells (right). Numbers indicate averaged gMFI values of two technical replicates from each experiment (single readout for the unstained condition). Cells were stained with DL550-labeled anti-FcRn Fab and an irrelevant Fab (isotype control) at pH 6.0 in living cells for FcRn surface expression (top panels), or after fixation and permeabilization for total FcRn expression (bottom panels). A representative experiment from two independent ones is shown. (B) Dose-response curves of hAK23-IgG4 Fc variants WT, DHS, IHH, and hIgG4 binding to human FcRn obtained by ELISA at pH 6.0 (upper) and pH 7.4 (lower). Solid lines with filled symbols represent data for non-labeled antibodies, and the dotted lines with empty symbols for fluorescently labeled antibodies. Increasing amounts of hAK23-IgG4 Fc variants (0.001 nM to 60 nM, except for the DHS variant: 0.0001 nM to 6.67 nM) were captured on immobilized biotinylated human FcRn, followed by detection of the bound hAK23 by an HRP-conjugated anti-human IgG-Fc F(ab')2 antibody. Data points represent the averaged OD_{450} values of two technical replicates from one representative experiment. Two independent experiments were performed. No statistical analysis was performed.

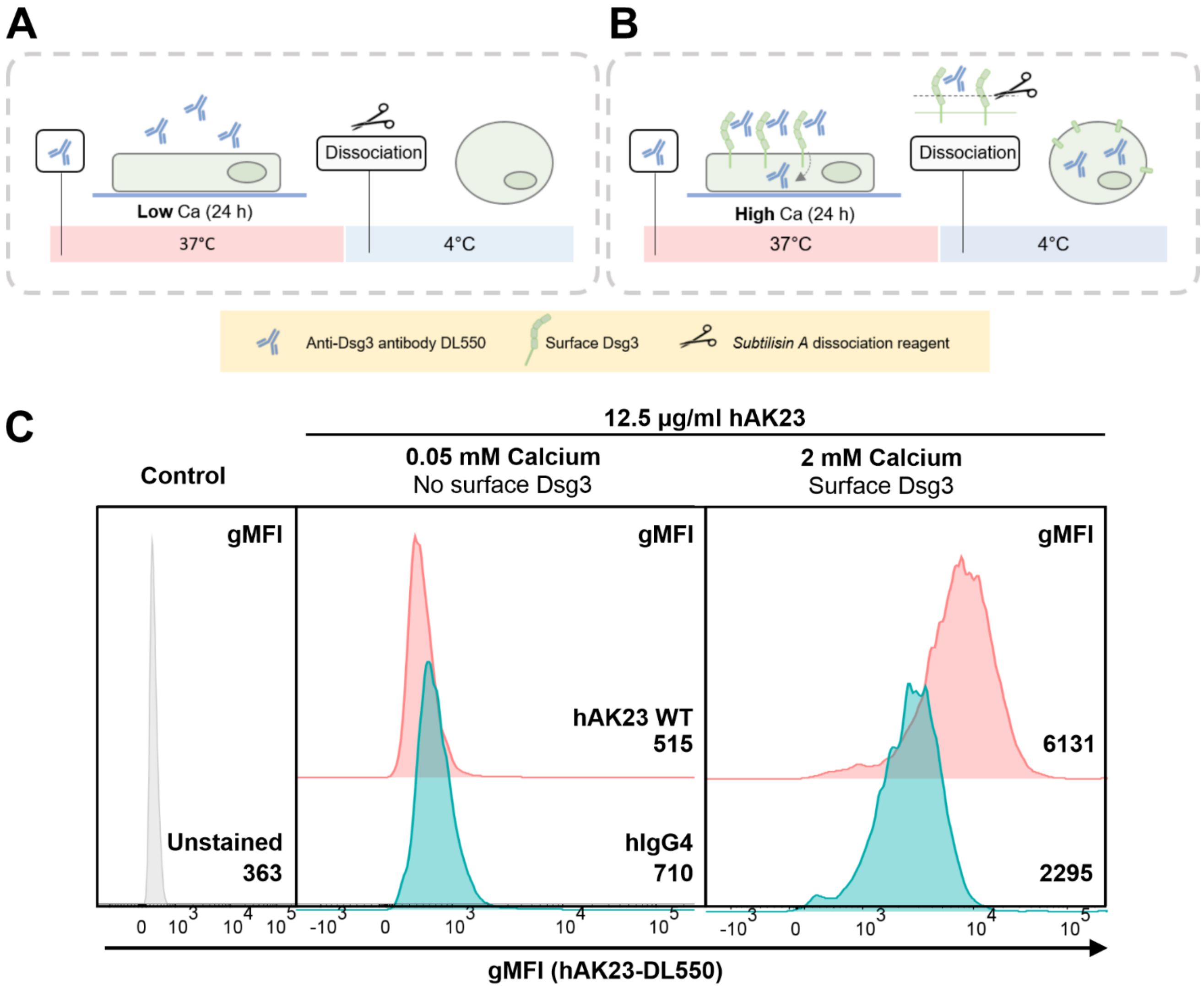

Supplementary Figure S3. Presence of Dsg3 on the cell surface is required for the uptake of anti-Dsg3 antibodies. (A) and (B): Schemes representing the experimental setup of the intracellular accumulation experiment under (A) low (0.05 mM) and (B) high (2 mM) calcium conditions. (C) Representative flow cytometry histograms of an intracellular accumulation experiment under 0.05 mM and 2 mM calcium conditions for comparison of the signals of DL550-labeled hAK23-IgG4-WT and hIgG4 in hTert cells. The cells were loaded for 24 h with 12.5 µg/ml hAK23-IgG4-WT or hIgG4 under culture conditions, washed extensively, and analyzed by flow cytometry. Numbers indicate the average gMFI values of two technical replicates (one replicate for unstained control).

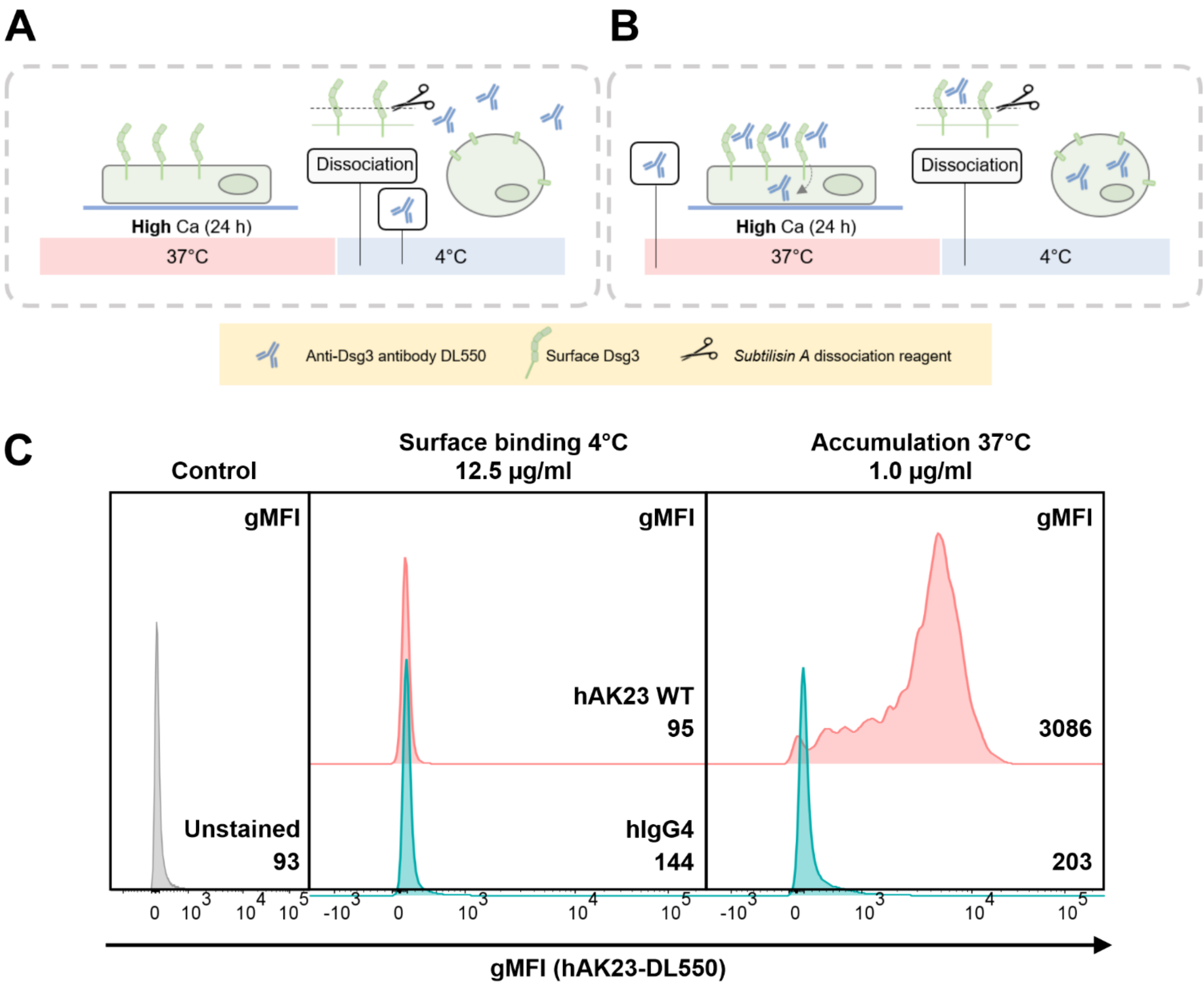

Supplementary Figure S4. Cell dissociation procedure prior to flow cytometry removes surface Dsg-3. Schemes representing the experimental setup of the surface Dsg3 binding (A) and the intracellular accumulation (B) experiments. (C) Representative flow cytometry histograms of surface Dsg3 and intracellular accumulation (total Dsg3), obtained by comparing the signals of DL550-labeled hAK23-IgG4-WT and hIgG4 in hTert cells. Cells were loaded with the antibodies for 15 min on ice (12.5 µg/ml, surface Dsg3), or for 24 h under culture conditions (1.0 µg/ml, total Dsg3), washed extensively and analyzed by flow cytometry. Numbers indicate gMFI values of one sample.

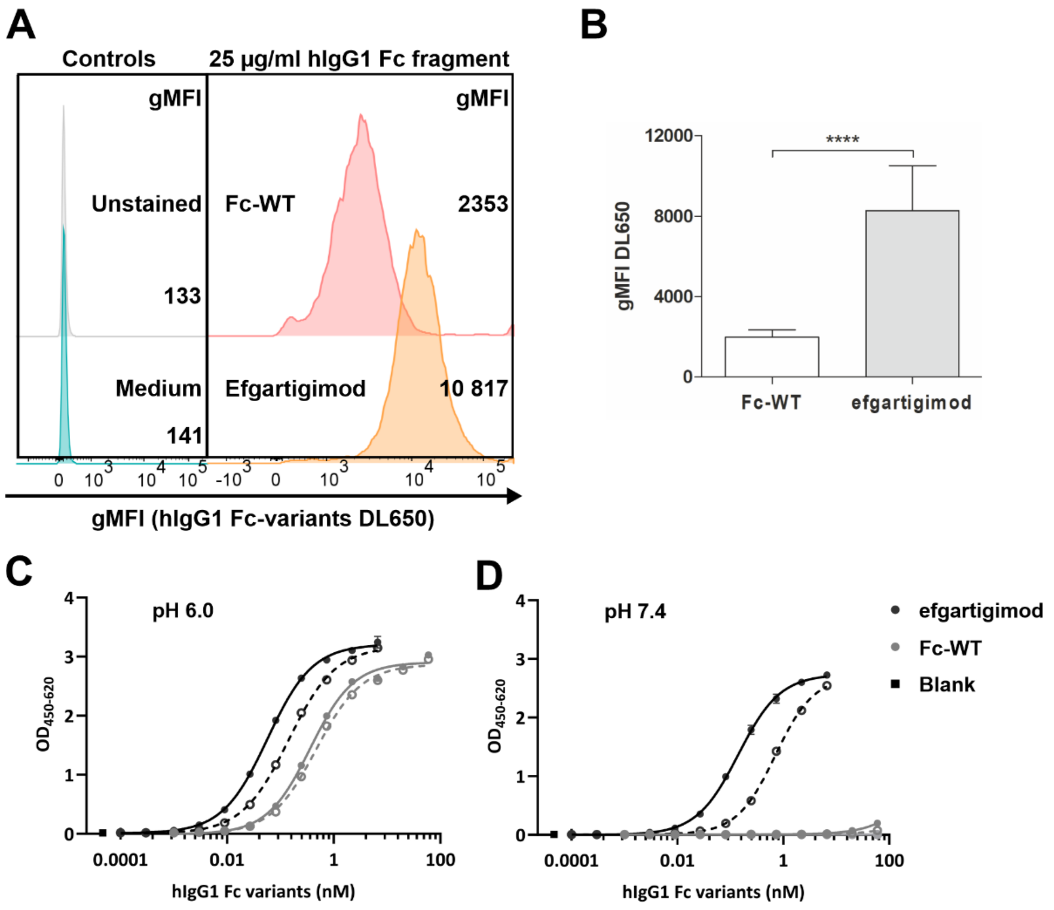

Supplementary Figure S5. Efgartigimod accumulates more efficiently in keratinocytes than a WT Fc fragment. (A) Representative flow cytometry histograms of an intracellular accumulation experiment comparing the signals of equally DL650-labeled efgartigimod and a human Fc fragment with a WT Fc region (Fc-WT-hIgG1) in hTert cells. Numbers indicate averaged gMFI values of two technical replicates from the same experiment (one replicate for unstained condition). Cells were loaded with the Fc fragments (25 µg/ml) for 24 h under culture conditions, washed extensively, and analyzed by flow cytometry. (B) Intracellular accumulation as mean gMFI from three independent experiments is shown. At least 4200 events in the live gate were acquired. Statistical analysis was performed on log-scale using a non-parametric t test assuming equal variances, and statistically significant differences are indicated by asterisks. **** = $p \leq$ 0,0001. (C) and (D): Dose-response curves of hIgG1 Fc-fragment variant binding to human FcRn, obtained by ELISA at pH 6.0 **(**C) and pH 7.4 **(**D**)**. Increasing amounts of Fc-WT (0.001 nM to 60 nM) or efgartigimod were captured on immobilized, biotinylated human FcRn, followed by detection of the bound Fc fragments by an HRP-conjugated anti-human IgG-Fc F(ab')2 antibody. Solid lines with filled symbols represent data for non-labeled antibodies and largely overlap with the dotted lines with empty symbols, representing data for the fluorescently labeled antibodies. The averaged OD_{450} values of two technical replicates from two independent experiments are shown.
